# Spatial diffusion of Zika fever epidemics in the Municipality of Salvador-Bahia, Brazil, in 2015-2016: does Zika fever have the same spread pattern as Dengue and Chikungunya fever epidemics?

**DOI:** 10.1590/0037-8682-0563-2019

**Published:** 2020-04-03

**Authors:** Laís Santos Santana, Jose Ueleres Braga

**Affiliations:** 1 Fundação Oswaldo Cruz, Escola Nacional de Saúde Pública Sergio Arouca, Programa de Pós-Graduação Stricto Sensu em Epidemiologia em Saúde Pública, Rio de Janeiro, RJ, Brasil.; 2 Fundação Oswaldo Cruz, Escola Nacional de Saúde Pública Sergio Arouca, Departamento de Epidemiologia e Métodos Quantitativos, Rio de Janeiro, RJ, Brasil.; 3 Universidade do Estado do Rio de Janeiro, Instituto de Medicina Social, Rio de Janeiro, RJ, Brasil.

**Keywords:** Zika, Chikungunya, Dengue, Arboviruses, Spatial diffusion

## Abstract

**INTRODUCTION:**

The recent emergence and rapid spread of Zika and Chikungunya fevers in Brazil, occurring simultaneously to a Dengue fever epidemic, together represent major challenges to public health authorities. This study aimed to identify and compare the 2015-2016 spatial diffusion pattern of Zika, Chikungunya, and Dengue epidemics in Salvador-Bahia.

**METHODS:**

We used two study designs comprising a cross-sectional-to-point pattern and an ecological analysis of lattice data. Residential addresses involving notified cases were geocoded. We used four spatial diffusion analysis techniques: (i) visual inspection of the sequential kernel and choropleth map, (ii) spatial correlogram analysis, (iii) spatial local autocorrelation (LISA) changes analysis and, (iv) nearest neighbor index (NNI) modeling.

**RESULTS:**

Kernel and choropleth maps indicated that arboviruses spread to neighboring areas near the first reported cases and occupied these new areas, suggesting a diffusion expansion pattern. A greater case density occurred in central and western areas. In 2015 and 2016, the NNI best-fit model had an S-curve compatible with an expansion pattern for Zika (R^2^ = 0.94; 0.95), Chikungunya (R^2^ = 0.99; 0.98) and Dengue (R^2^ = 0.93; 0.99) epidemics, respectively. Spatial correlograms indicated a decline in spatial lag autocorrelations for the three diseases (expansion pattern). Significant LISA changes suggested different diffusion patterns, although a small number of changes were detected.

**CONCLUSIONS:**

These findings indicate diffusion expansion, a unique spatial diffusion pattern of Zika, Chikungunya, and Dengue epidemics in Salvador-Bahia, namely. Knowing how and where arboviruses spread in Salvador-Bahia can help improve subsequent specific epidemic control interventions.

## INTRODUCTION

In recent years, the emergence of arboviruses in different territories and countries of the Americas, including Brazil, has been observed^1^. The most recent arbovirus identified in Brazil was Zika fever in 2015, which triggered an epidemic of undetermined exanthematous disease in the northeast region^2^. 

The disease presented in most individuals with a mild and benign evolution; however, a concentration of Guillain-Barré syndrome cases generated hypotheses that the Zika virus infection was associated with neurological and autoimmune complications in Brazil, as had also been suspected in French Polynesia in 2013^3^ ^,^ ^4^. Moreover, a considerable increase in the number of reported cases of microcephaly in newborns provided evidence supporting a connection between Zika fever and congenital neurological malformations^5^.

Prior to the sudden rise in Zika fever cases, Chikungunya fever emerged in Brazil in 2014^6^. Along with its severe articular pain characteristic, severe manifestations such as neurological, cardiac, renal, and ocular complications have been reported to be associated with the disease^7^.

These two arboviruses emerged amidst an endemic Dengue fever circulation present since 1986, involving four viral serotypes and considered one of the most prevalent infectious diseases. A triple epidemic in 2016 resulted in approximately 2 million reported cases^8^ ^,^ ^9^.

These arboviruses have several characteristics in common; transmission occurs through the same vector, namely, the Aedes aegypti mosquito, which is abundant throughout Brazil, and these arboviruses are derived from very close families that have similarities in signs and symptoms, making diagnosis difficult^10^ ^,^ ^11^. The vector control measures and the main strategy for disease control were inefficient and favored the propagation of arboviruses in Brazil^12^.

Given this situation, an analysis of the geographical processes of spatial diffusion can be used for monitoring epidemics and planning interventions, since it enables an understanding of how and where diseases propagate through space and in the course of time^13^. In 2014, Sant-Julien stated that the process of spatial diffusion is the action, or the result of an action of propagating a phenomenon homogeneously in a system, whatever the force that drives the dispersion of this phenomenon^14^. The visualization of spatial patterns in data and the description of these patterns aims to predict changes and uses this information to guide the formulation of policies^15^.

In the most commonly used classification, it is stated that spatial diffusion can occur through four phenomena. The first is expansion, in which the phenomenon propagates from one place to the other, often intensifying in the region of origin. The second phenomenon is relocation, when the phenomenon propagates to new localities but when moving, leaves the areas where it originated. The third is contagion, dependent on direct contact and, therefore, strongly influenced according to distance (individuals in nearby regions have a much higher probability of contact than individuals in distant regions). Lastly, a hierarchical phenomenon is characterized by the occurrence of diffusion respecting an ordered sequence of classes or places, as from a large metropolitan center to remote villages^16^.

A very limited number of studies have sought to specifically analyze the spatial diffusion of arboviruses, with an emphasis on investigating Dengue fever in the state of Bahia, Brazil^17^ ^,^ ^18^. This study aimed to identify and compare the 2015-2016 spatial diffusion pattern of Zika, Chikungunya, and Dengue epidemics in Salvador-Bahia.

## METHODS

**FIGURE 1:**
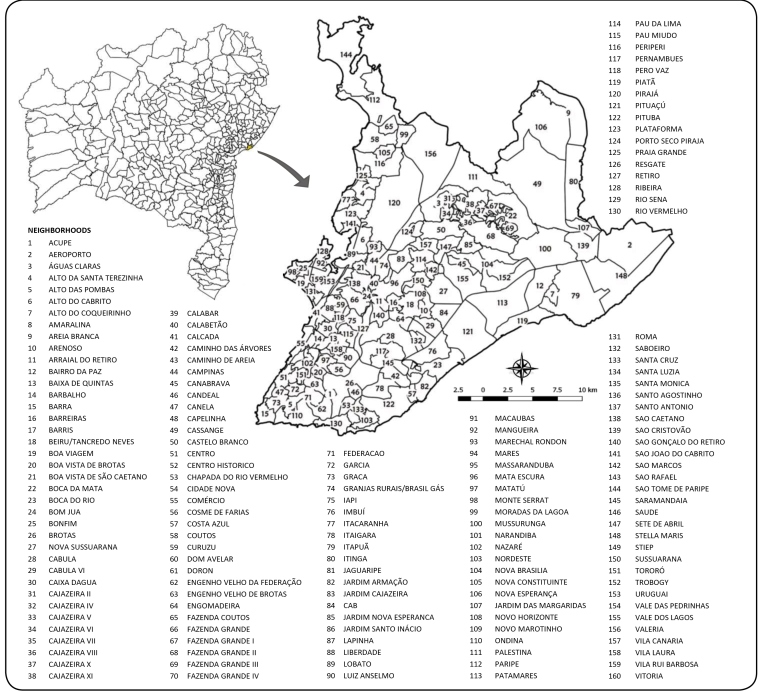
Location of the municipality of Salvador in the state of Bahia and its territorial division.

Two study designs were used, a sectional study for the point spatial data, and an ecologic study for the area data. The study area comprised the municipality of Salvador, the capital of state of Bahia which is the third most populous city in Brazil with an estimated population of 2,938,092 inhabitants in 2016^19^ and 163 neighborhoods (Figure 1)^20^.

In the data analysis, three island neighborhoods namely Ilha dos Frades, Ilha de Maré, and Ilha de Bom Jesus dos Passos were excluded, because we considered that, through discontinuity with the territory, the processes that act for spatial diffusion of arboviruses could have differed. Centro Administrativo da Bahia (CAB) and Aeroporto were also excluded since they are not residential neighborhoods, making 5 of 163 neighborhoods excluded from the study.

The study period covered the epidemic waves that occurred in the years 2015 and 2016. For Zika fever, the first wave comprised epidemiological weeks (EW) 15/2015 to 52/2015, and for Chikungunya and Dengue fever, the first wave comprised the EWs from 01/2015 to 52/2015. For the three arboviruses, we considered the second wave comprised the EWs from 01/2016 to 52/2016.

Confirmed cases were used, as notified in the *Sistema de Informação de Agravos de Notificação-SINAN* of the Brazilian Ministry of Health. Residential addresses for each case were georeferenced to generate punctual spatial data using three automated geocoding application programming interfaces to obtain a greater number of georeferenced cases: Google Maps, Bing Maps, and Open Street Maps.

For aggregated data, we opted for smoothed incidence rates (empirical Bayesian) because of the large variation of gross rates that would have strongly influenced the analyses. The number of people living in the neighborhoods of Salvador in 2015 was obtained from estimates according to the Brazilian Institute of Geography and Statistics (IBGE), based on a 2010 census, and a linear interpolation was performed to estimate the population for the year 2016^19^ ^,^ ^21^. Cartographic digital meshes of the municipalities and the neighborhoods were obtained from the IBGE.

An analysis of spatial diffusion patterns was performed using various techniques that have been previously applied in other studies, namely visual inspection of the sequential kernel and choropleth map, spatial correlogram analysis, local spatial autocorrelation (LISA) changes analysis, and the nearest neighbor index (NNI) modeling.

For visual inspection of sequential maps, 30 choropleth maps and 30 kernel maps were generated with data from every three EWs of Zika fever. For Chikungunya and Dengue fevers, 35 thematic and 35 kernel maps were generated.

Kernel estimation is a method for smoothing point events through inserting a continuous surface over each point and requires a bandwidth of the region of influence. A smoothed surface is displayed on the territory representing the levels of case intensity^22^. Kernel maps were developed with a bandwidth of 2500 m, using QGIS software^23^.

Choropleth maps display levels of the same attribute per area at each time range for sequential demonstration of the distribution of the disease. The maps were constructed using five classes of incidence rates using QGIS software^23^.

In the modeling of the NNI, the ratio of the nearest neighbor, or R, is calculated by dividing the mean of the distances of the nearest points in a point pattern by the average of the distances of a random distribution of the same number of points in the same area. R is calculated cumulatively whenever a new case is diagnosed. The Rs are presented as a function of time and modeled with a set of regression curves to find the one that best fits the distribution. The results were interpreted according to Lee et al. (2014)^24^. Their approach indicates that the processes of diffusion through contagion and expansion are better adjusted using an inverse curve, and the hierarchical and relocation processes are better modeled using a cubic curve.

In our study, linear, logarithmic, inverse, quadratic, cubic, power, compound, S-curve, logistic, growth, and exponential regression curves were constructed using the “curvefit” function of Stata software^21^. These curves were compared to the annual R curves plotted against time so that the best fit was identified with the coefficient of R².

For the analysis of the spatial correlograms, the values ​​corresponding to Moran's I (global autocorrelation indicators) were calculated using an adjacency matrix (determining the spatial autocorrelation) and plotted against the spatial lags referring to the distance intervals. The correlograms were developed for each arbovirus annually, using “R statistical software”^25^ in conjunction with QGIS software^23^.

The incidence of each arbovirus was considered autocorrelated in space using definitions proposed by Lam et al. (1996)^26^, in which a propagation by contagion model is considered when the correlogram shows a tendency to autocorrelation in a soft decline, pointing to distance as a principal factor in determining the similarity of incidence rates. A hierarchical diffusion is suggested when the trajectory decreases and then rises, forming a V-shaped curve, indicating that distant areas show similarity in rates and that the diffusion then occurs in jumps.

To apply the technique of change in local autocorrelation, the Cohen and Tita (1999) study was used as a reference^27^. LISA indicators consist of the decomposition of Moran's I^28^.

The values ​​of a variable for a location and its neighboring areas provide the most basic representation of local spatial associations. Each Local-Neighbor (LN) pair consists of standardized levels of a variable in the local spatial unit L and in neighboring spatial units N. Each element of the pair is low (L) or high (H) relative to the local and neighbor value distributions in all observations^27^.

In a scatterplot, pairs in which both local and neighbor values ​​are above their respective averages fall in the upper right quadrant (HH), or into the lower left quadrant (LL). When they differ, pairs fall into the HL or LH quadrant. 

For a dynamic view of the process, Cohen and Tita (1999)^27^ suggested analyzing changes in local-neighbor pair levels over time, looking for evidence of diffusion that involves the propagation of high rates (or low rates) to other spatial units. The authors claimed that the types of pair changes in successive periods were compatible with a different type of diffusion, and allowed for a determination of the mechanisms behind the change corresponded to each type of diffusion.

LISA values were calculated using GeoDa software^29^, based on the incidence rates of each arbovirus, with reference to periods of three EWs. Analyzed annually, changes in the predominant local autocorrelation levels determined the spatial diffusion type for each arbovirus.

## RESULTS

**FIGURE 2:**
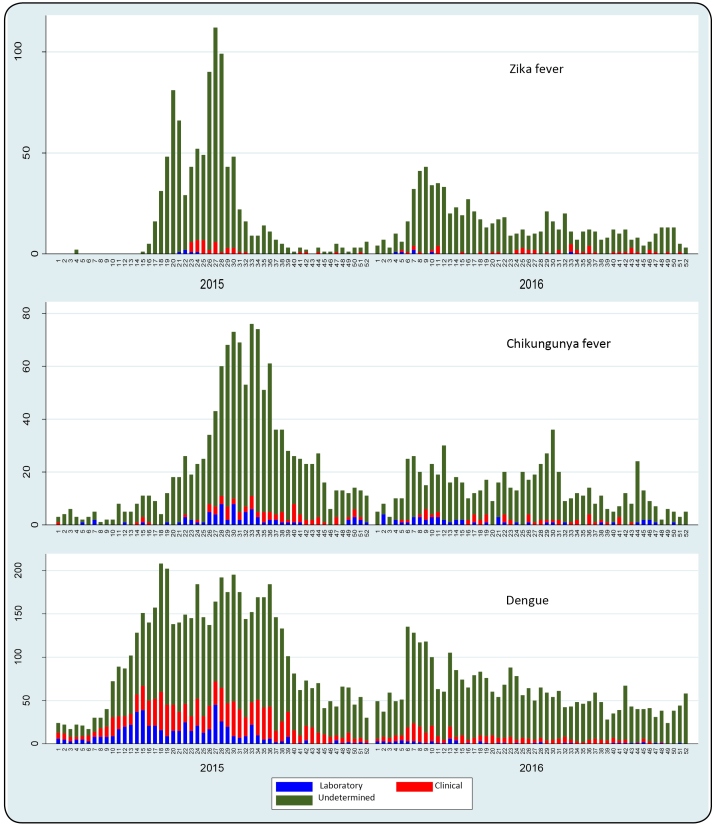
Distribution curves of reported cases of Zika fever, Chikungunya fever and Dengue in Salvador, 2015-2016.

The distribution curve of Zika fever cases showed two epidemic waves. The first occurred between EW 15 and EW 41 in 2015, with an epidemic peak at EW 27 (112 cases). The second less significant epidemic, occurred between EW 01 and EW 22 in 2016, with a higher concentration at EW 9 (43 cases) (Figure 2).

The distribution curve of Chikungunya fever cases shows an epidemic wave in 2015, with a higher case concentration between EW 26 and EW 38, and an epidemic peak at EW 33 (76 cases). Waves of lesser intensity were identified throughout 2016.

The distribution curve of Dengue fever cases shows a large epidemic wave that covered the entire year in 2015, with a higher concentration of cases between EW 14 and EW 39. During 2016, there was a regular level of reported cases.

The majority of reported cases involving the three arboviruses lacked confirmatory diagnostic criteria (undetermined), and diagnosis was presumably based on clinical-epidemiological criteria, as laboratory confirmation is usually performed at the beginning of epidemics to confirm autochthony.

The georeferencing of Zika fever cases in Salvador resulted in 1,780 (93%) georeferenced addresses for the 1,914 cases confirmed between 2015 and 2016. These cases were organized according to the date of first symptoms, and 32 cases were discarded due to the date of first symptoms field having been incorrectly completed. In total, 1,748 cases were considered in the statistical analysis and, of these, there were 937 cases in 2015 and 811 in 2016. Of 2,120 Chikungunya fever cases, 1,974 (93%) were georeferenced, 38 were discarded, and we used 1,210 cases from the year 2015 and 726 from the year 2016 in the statistical analysis. Of 9,302 cases of confirmed Dengue fever, 8,938 (96%) were georeferenced, 301 discarded, and we used 5,449 cases for the year 2015, and 3,188 for the year 2016.

**FIGURE 3:**
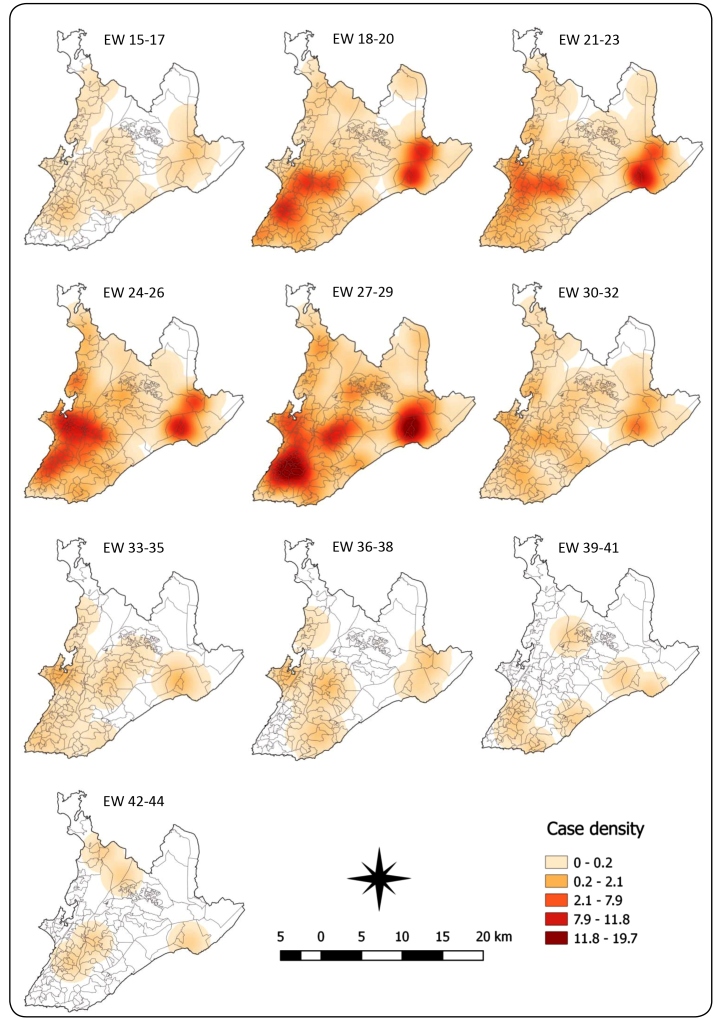
Kernel maps for analysis of the spread of Zika fever in Salvador, EW 15-44/2015 (the maps of the last EW were hidden due their similarities).

Zika, Chikungunya, and Dengue fever cases were recorded in approximately 95% of neighborhoods analyzed in the municipality of Salvador. The analysis of kernel maps (Figure 3) and choropleth maps suggested that the spatial diffusion process of Zika fever occurred through expansion, as did Dengue and Chikungunya fevers. Some areas of the municipality, mainly in the west/southwest region, showed an emergence of cases that then intensified and reached nearby regions.

Regarding Zika fever, the northeast of the municipality also presented a greater concentration of cases in 2015. In Chikungunya and Dengue fevers, the central region was highlighted. In 2016, the diseases appeared to have occurred with less intensity, with a greater concentration of cases observed in the central region.

For Zika fever, 144 and 311 R-values ​​were generated for the years 2015 and 2016, respectively. For Chikungunya fever, 285 and 303 R-values ​​were generated for the years 2015 and 2016, respectively. For Dengue fever, 357 and 414 R-values were generated for the years 2015 and 2016, respectively.

**FIGURE 4:**
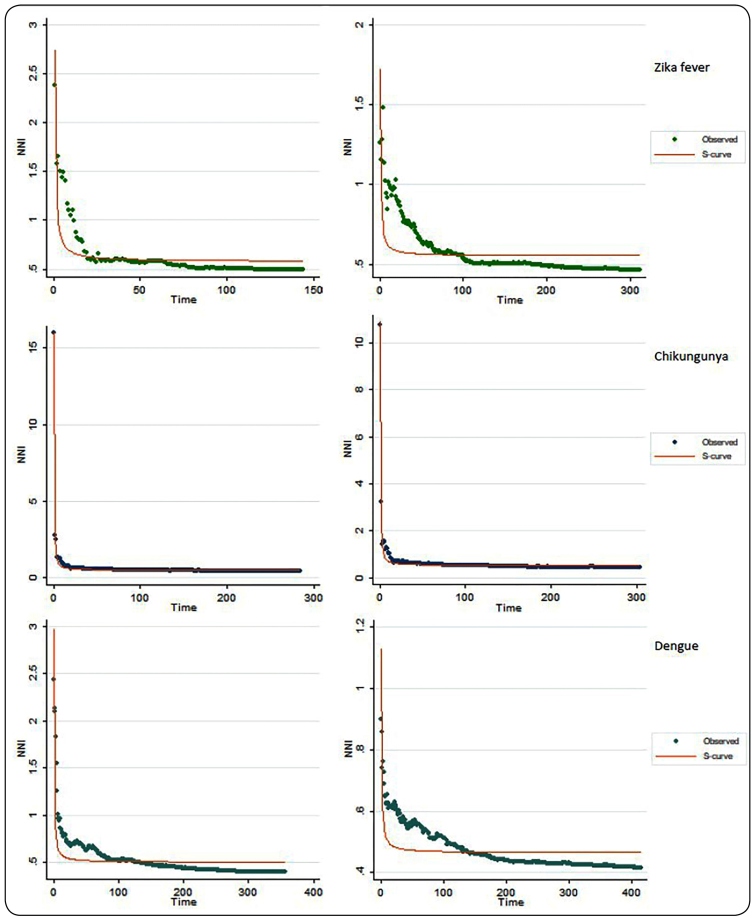
Regression curves of better fit to Nearest Neighbor Index (NNI) curves of Zika fever, Chikungunya and Dengue in Salvador, 2015-2016.

The regression curve that best fit the R curves was the S-curve (Figure 4), with R^2^ 0.94;0.95 (Zika fever), R^2^ 0.99;0.98 (Chikungunya fever), and R^2^ 0.93;0.99 (Dengue fever) in 2015 and 2016, respectively; therefore, showing a process of diffusion through expansion.

**FIGURE 5:**
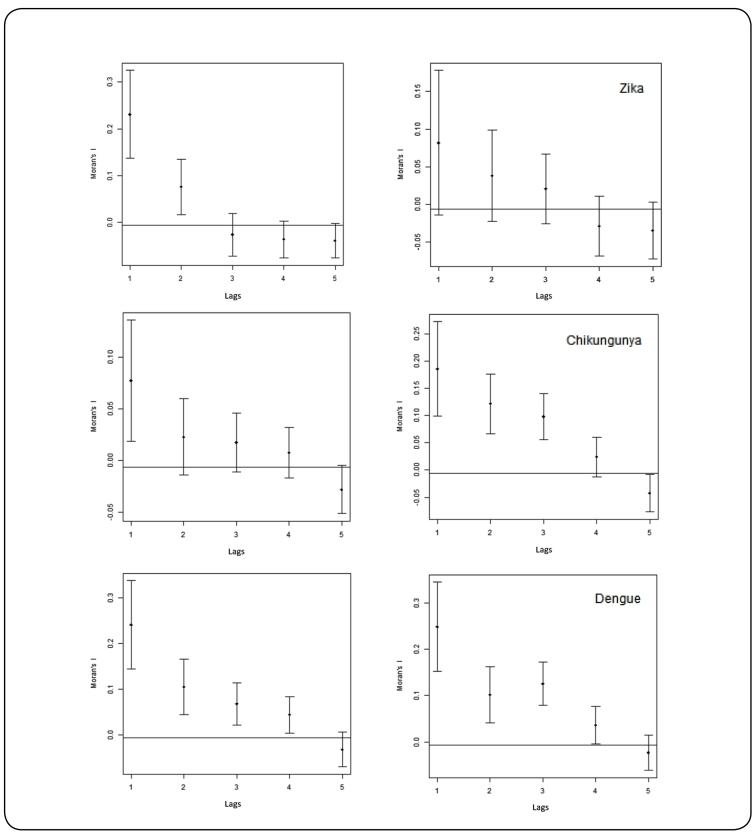
Spatial correlograms of Zika fever, Chikungunya and Dengue in Salvador, 2015 - 2016.

The correlograms of the three arboviruses show a smooth decline in spatial autocorrelation as the distance between spatial lags increases (Figure 5). This decline shows that expansion diffusion was the dominant factor for the spread of Zika, Chikungunya, and Dengue fevers in the municipality of Salvador. That is, the municipality presented areas with high incidence rates and neighboring areas that had lower incidence rates, indicating that these diseases progressively spread from one region to their neighboring regions.

In the analysis of LISA changes, LISA values ​​were generated with reference to periods of three EWs, for each of the 160 neighborhoods within the Salvador municipality. Analyzing the changes in these values, 19 of 1,280 LISA changes were significant for Zika fever in 2015 and 34 of 2,400 LISA changes were significant for Zika fever in 2016. The local autocorrelation changes characteristic of expansion diffusion[14] were predominant in 2015 and hierarchical diffusion[20] in 2016. Relocation diffusion was considered likely here, along with hierarchical diffusion, due to similarity in the characteristics of the processes that occurred in jumps.

For Chikungunya fever, 42 out of 2,400 LISA changes were significant in 2015 and 19 out of 2,080 LISA changes were significant in 2016. In two years, the type of spatial diffusion was mixed because the number of changes related to the expansion and hierarchical diffusion were equal in 2015[21;21], and similar in 2016[10;19].

For Dengue fever in 2015, 62 out of 2,560 LISA changes were significant and 79 out of 2560 changes were significant in 2016. The number of changes characterizing the hierarchical diffusion process[34] surpassed the expansion[28] in 2015 but was very close in 2016[39;40] with diffusion mixed in this year. 

## DISCUSSION

The analysis of the spatial diffusion of Zika fever, Chikungunya fever, and Dengue fever in Salvador demonstrated that the propagation of the three arboviruses occurred through expansion in the two years of study.

In 2015, Zika and Chikungunya fevers broke out in Salvador and, as expected, due to population susceptibility and abundance of the main vector, *Aedes aegypti*, the arboviruses caused an explosive number of cases.

Although Dengue fever had already established in this period in Salvador, it showed a significant increase in notifications, which was observed throughout Brazil. Experts attribute this increase to the reemergence of the DENV1 serotype in 2013 and to a reduced DENV4 circulation, taking into account that the presence of the four serotypes of the Dengue virus in Brazil often trigger epidemics of the disease after alternating or replacing the predominant serotype^30^ ^,^ ^31^. The antigenic diversity of the Dengue virus is a contributing factor to the continuous occurrence of the disease, since multiple sequential infections may occur due to the lack of cross-immunity between virus serotypes^32^.

Our study demonstrated that the three diseases emerged and intensified in some areas of Salvador, and initially expanded to nearby sites through the expansion diffusion process. This finding is compatible with the dynamics of infectious diseases, where transmission is more likely to occur among individuals who are closely connected in space and time^30^.

For the year 2016, our results indicated that the three arboviruses had spread through expansion, although with less intensity. The progressive decrease in the number of new cases was consistent with the dynamics of the arboviruses in the second year of propagation because, although the number of new cases continued to intensify in places with greater population density, a decrease in the susceptible population in some areas led to a lower occurrence of diseases in the greater part of Salvador.

The diffusion through expansion model was identified by Barreto et al. (2008)^17^ in relation to the first Dengue epidemic in Salvador in 1995, during the emergency period of this arbovirus. Lee et al. (2004)^24^ identified the same pattern concerning the spatial diffusion of Dengue fever between 2003 and 2008 in Taiwan.

Morato et al. (2015)^18^ identified a different pattern in their study on the spatial diffusion of Dengue fever in the municipality of Jequié, Bahia, in 2009, where they showed that the disease epidemic occurred initially through expansion and later through relocation.

In addition to the identification of the spatial diffusion processes, analysis of kernel maps indicated that the three diseases had a main focus of transmission in an area that extended from the west/southwest region of the municipality, where administrative districts are concentrated, to the central region, with both regions having higher population density and social interaction networks. This area sustained a high number of cases throughout almost the entire study period, possibly due to the higher concentration of susceptible cases compared to other areas.

Areas with high population density and economic development that can act as transmission focuses reinforce the pattern of diffusion through expansion as a characteristic of communicable diseases, reaching a greater number of people who are interacting in close quarters^15^.

These areas are compatible with the epicenter of the first Dengue fever epidemic, also occurring in Salvador, as reported by Barreto et al. (2008)^17^. This similarity suggests that, if interventions had been directed to those areas of previous Dengue fever epidemics, the expansion of arboviruses and the recent triple epidemic could have been minimized.

Among the visualization methods, the kernel maps provided a better appreciation of the spatial diffusion than the choropleth maps, because they exhibited areas with a higher density of cases where the risk of arbovirus transmission may have been higher due to prevalent cases.

Choropleth maps were more difficult to interpret because of excessive variation in incidence rates, a phenomenon less frequent in large populations and more important when small numbers of cases are related to small populations. The color scale has been reported to be also very sensitive to variations, which hinders interpretation^15^ ^,^ ^33^. Apart from this, the diffusion processes were evaluated in conjunction with other techniques used.

Despite a high degree of subjectivity, the use of visual presentation methods combined with statistical techniques can make quantitative results easier to interpret.

Among the statistical methods used, the LISA changes analysis technique produced a different result for the three arboviruses compared to the other techniques. However, the LISA result should be interpreted with caution because of the small proportion of statistically significant changes found in our study (<3%).

The spatial correlograms presented similar configurations. The NNI analysis technique appeared to be the most robust method of analysis as it was based on a statistical approach that used daily information, was modeled using regression curves, and could be compared to other analyses previously simulated by Lee et al. (2008)^24^.

The main limitation of this study is that the surveillance data may have contained a poor diagnostic classification in reports, possibly due to the rapid emergence of arboviruses and similarity in the symptoms when defining the diagnoses according to clinical epidemiological criteria. Simultaneous circulation of the three arboviruses was a factor that may have compromised the investigation of some of the diseases and led to more intense investigation of others. Since most cases were asymptomatic, we considered that a substantially lower number of cases might have been reported. It is likely that diagnostic cross-classification errors occurred during the simultaneous triple epidemic, but it is difficult to evaluate the magnitude of such bias. It might be that the cross-diagnosis brings the specific spatial diffusion patterns close to that observed for Dengue. This would obscure specific characteristics that might happen for Zika and Chikungunya.

Despite this limitation, the combination of statistical and visualization techniques appeared to allow the analysis of spatial diffusion. This information should allow the implementation of more effective control programs by directing prevention and control strategies to specific areas and periods, as recommended by Cliff et al. (1981)^16^.

Our study of the spatial diffusion of Zika, Chikungunya, and Dengue arboviruses in in Salvador-Bahia, in the period of triple epidemics between 2015 and 2016, enabled an identification of the propagation process of these arboviruses, indicating that the diffusion pattern of the Zika fever epidemics was the same as that of the Dengue and Chikungunya fever epidemics. During the two years of study, these diseases spread through diffusion expansion. This knowledge on how and where arboviruses spread in Salvador in a triple epidemic situation provides important information for improving epidemiological surveillance, both in terms of monitoring cases and in terms of targeting interventions for disease control.
